# Development of palliative care clinical practice guidelines and referral care pathways for primary care practitioners in Pakistan

**DOI:** 10.1186/s12904-024-01438-y

**Published:** 2024-05-01

**Authors:** Syeda Amrah Hashmi, Russell Seth Martins, Annum Ishtiaq, Nashia Ali Rizvi, Mohsin Ali Mustafa, Alina Pervez, Ayra Siddiqui, Syeda Fatima Shariq, Sarah Nadeem, Adil H. Haider, Muhammad Atif Waqar

**Affiliations:** 1 Center for Clinical Best Practices, Clinical and Translational Research Incubator (CITRIC), Aga Khan University Hospital, Karachi, 74800 Pakistan; 2 Section of Palliative Medicine, Department of Oncology, Aga Khan University Hospital, Karachi, 74800 Pakistan; 3 Medical College, Aga Khan University, Karachi, 74800 Pakistan; 4 Section of Endocrinology, Department of Medicine, Aga Khan University Hospital, Karachi, 74800 Pakistan

**Keywords:** Palliative Care, Clinical Practice Guideline, Referral, Pakistan

## Abstract

**Background:**

Despite a large burden of life-limitingillness, there exists a dearth of services of palliative care in Pakistan. International guidelines have questionable applicability in Pakistan due to the socioeconomic differences. We generated a protocol describing the process of developing comprehensive palliative care guidelines and palliative care referral pathways for primary care practitioners to adopt in Pakistan.

**Methods:**

A GRADE-ADOLOPMENT approach with modification has been employed to create guidelines for a Pakistani context. The “*National Comprehensive Cancer Network Guidelines Insights: Palliative Care, Version 2.2021”* was used as the source guideline. Recommendations from the source guideline were reviewed by two local palliative care specialists to either “*Adopt*,” “*Adapt”* or “*Exclude*”. The finalized recommendations were incorporated into the local palliative care guideline. Clinical diagnosis and referral pathways were made from the finalized guideline. Any gaps in management found in the pathways were filled by taking existing recommendations from other credible guidelines.

**Results:**

Twenty-seven recommendations were adopted without modification. No recommendations were deemed to be adapted and 15 were excluded. The referral care pathways created were reflective of the local guideline and included elements of initial assessment, preliminary management, reassessment, and referral. 6 additional recommendations were made.

**Conclusion:**

The described clinical practice guidelines and primary care clinical referral pathways will aid to standardize palliative care provision in Pakistan. These can be used by other resource constrained settings to develop guidelines within their own local context.

**Supplementary Information:**

The online version contains supplementary material available at 10.1186/s12904-024-01438-y.

## Background

Palliative care aims to reduce suffering, provide holistic support, encourage adaptive coping, and through the integration of physical, psychological, social, and spiritual healthcare alongside analgesic and symptomatic control to improve patients’ quality of life [1]. A multidisciplinary team, including physicians, nurses, psychologists, and physiotherapists is responsible for delivering it [1]. Patients with life-limitingdiseases, including end-stage organ diseases or cancer are the primary recipients of palliative care. The burden of global cancer is expected to increase by 47% by 2040 [2], with greater cancer-associated morbidity and mortality in lower-middle-income countries [3]. Pakistan, a country from South Asia classified as lower-middle-income, has a population of more than 220 million, with more than 175,000 new cancer cases in 2020 [4]. Despite this, alarmingly, palliative care services are practically non-existent in Pakistan, with only a handful of facilities across the entire country [5].

The principles delineated in best-evidence clinical practice guidelines (CPGs) serve as the bedrock of care for palliative patients. The majority of the widely utilized international guidelines were previously developed by high-income countries, particularly the United States of America, United Kingdom and other Western countries [6, 7] . and the United Kingdom [8]. These countries possess well-established palliative care facilities, and unsurprisingly, also lead global palliative care research efforts [9]. In lower-middle-income countries, palliative care is usually informed by such pre-existing, freely available guidelines, as lower-middle-income countries lack the financial ability, research capacity, and expertise to create local guidelines to meet their own unique needs. The lack of locally developed clinical practice guidelines represents an urgent problem for lower-middle-income countries including Pakistan, where the practice of palliative medicine may be expected to differ due to myriad reasons. These include a lack of palliative medicine specialists [5], out-of-pocket healthcare financing [10], poor health-seeking behavior and disease-related awareness [11–13], poorer socioeconomic and educational backgrounds [12, 13], and socio-religio-cultural influences [11, 14]. It is to account for differences such as the aforementioned, that it is generally recommended that even clinical practice guidelines of the highest quality should be tailored to suit the needs of the specific setting of their use [15].

**Fig. 1:**
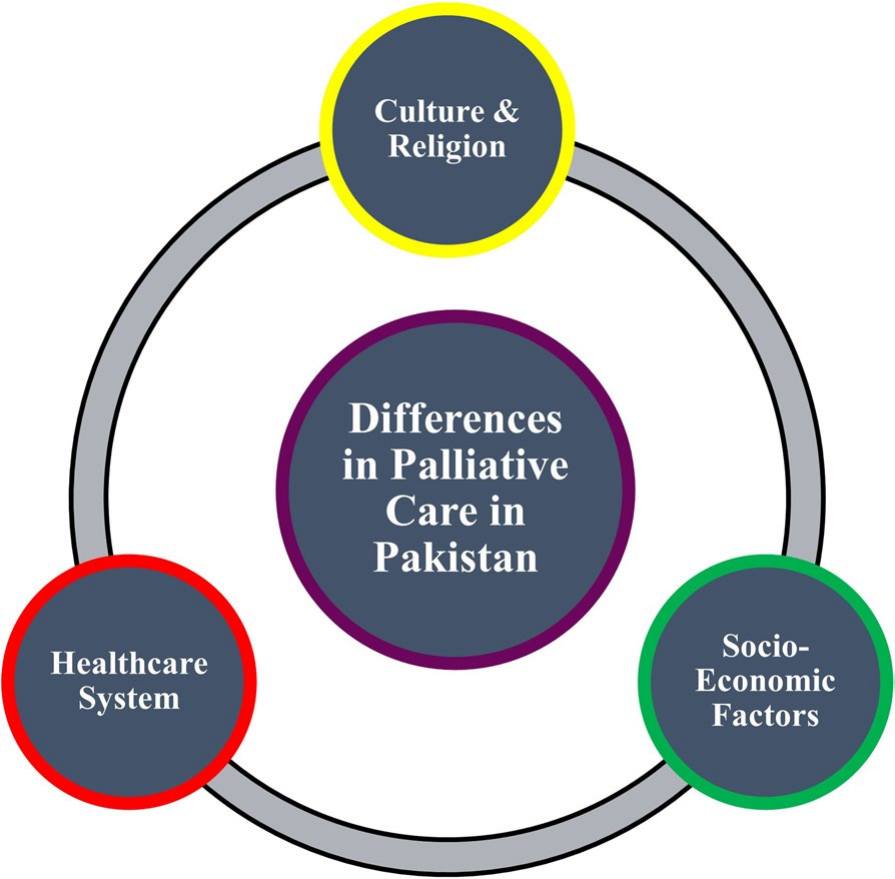
Possible sources of differences in palliative care in Pakistan

Currently, the Government of Pakistan’s Ministry of National Health Services Regulation and Coordination endorses the “*National Coalition for Hospice and Palliative Care Clinical Practice Guidelines”*for use in Pakistan [16]. Although this clinical practice guideline is substantiated with well-documented, high-quality research, it is of questionable applicability in the setting of a shortage of resources and infrastructure in Pakistan. For instance, Pakistan has less than ten palliative care facilities across the entire country [5, 17]. While at first glance this might seem like an issue that can be resolved with adequate medical infrastructure, the problem goes deeper (Fig. 1). It can be argued that, given the collectivist nature of Pakistani society, palliative centers in the country for inpatient admissions carry the risk of depriving patients from the strong family support system they find at home. Moreover, as Islam is the predominant religion in Pakistan, religious beliefs of patients and providers are also likely to manifest in different perspectives to palliative care than in the West [18–20]. Lastly, the acute lack of palliative care specialists in Pakistan [5] is unlikely to be met soon. In the interim, the next best suggested solution is to leverage the large workforce of general practitioners to provide basic palliative care services [21]. Thus, use or endorsement of any pre-existing clinical practice guidelines in Pakistan must take into consideration the differences in ground realities in the socio-economic and healthcare landscapes.

While lack of research and resources precludes the de novo creation of clinical practice guidelines in Pakistan, local context-specific guidelines can also be formed via a process known as “adolopment”. This recently introduced term combines adoption (using without modification), adaptation (using with relevant modifications), exclusion (omitting due to irrelevance), and de novo development [15]. The process of “adolopment” undoubtedly proves more judicious for guidelines in the context of a lower-middle-income country like Pakistan [15]. The GRADE (Grading of Recommendations Assessment, Development and Evaluation)-ADOLOPMENT approach is a previously described “adolopment” framework [15] that can be used to form a credible clinical practice guidelines in a resource-constrained setting like Pakistan. Evidence-to-decision (EtD) tables are utilized, allowing a panel of experts to use the best available evidence for consensus decision-making, to adapt recommendations deemed to require context-specific modification. EtD tables help judge context-specific appropriateness of individual recommendations across 12 criteria, which include acceptability, feasibility, equity, and cost-effectiveness, among others. Consensus changes to recommendations are incorporated into local guidelines.

Given the shortage of palliative care specialists in Pakistan, primary care practitioners can help provide palliative care by providing appropriate counseling, education, pharmacological interventions, screening, and, if needed, specialist referral [21]. This requires, as the bare minimum, credible clinical practice guidelines available to primary care practitioners across the country. Therefore, there is utmost necessity to develop local palliative care CPGs through a standardized and transparent process that utilizes existing available best-evidence clinical practice guidelines incorporating context-specific modifications. The implementation of these CPGs will cause a significant advancement in the healthcare system of Pakistan in achieving better access to palliative healthcare, delivered via GPs. Thus, we have aimed to formulate a local evidence-based CPG with referral pathways based on the GRADE-ADOLOPMENT process for palliative care in Pakistan with focus on cancer patients. This can also pave way for future similar efforts for non-malignancy related palliative care, which also represents a significant problem.

## Methods

### Study setting

This study was conducted at the CITRIC (Clinical and Translational Research Incubator) Center for Clinical Best Practices at the Aga Khan University Hospital, Pakistan. The Aga Khan University Hospital, a not‐for‐profit, private sector hospital in Pakistan, is recognized as the country’s preeminent healthcare and biomedical research facility [22].

**Fig. 2:**
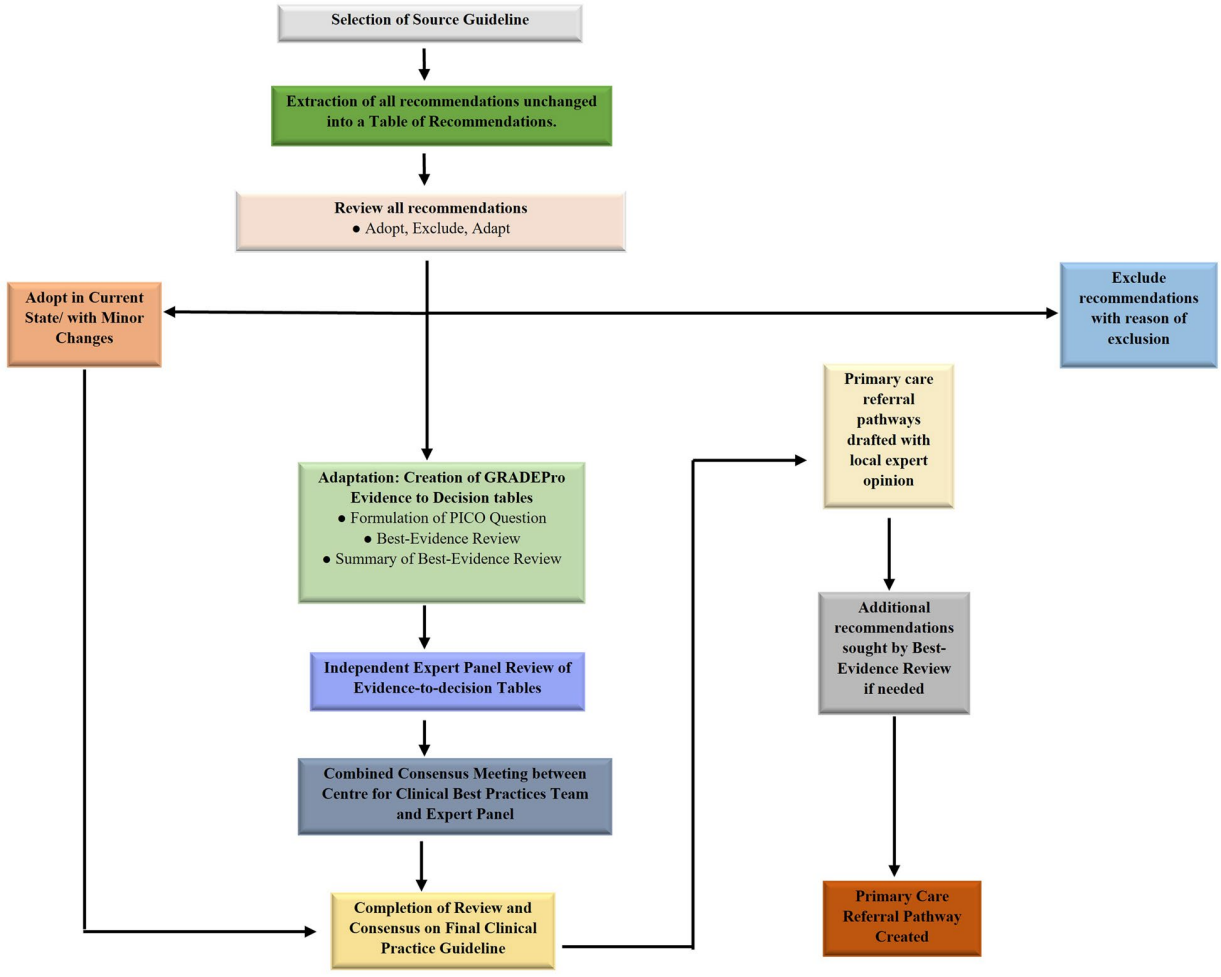
GRADE ADOLOPMENT and primary care referral pathway development

The Center for Clinical Best Practices at Aga Khan University Hospital is working on the development and adaptation of evidence-based guidelines and care pathways for the improvement and standardization of healthcare in Pakistan and other lower-middle-income countries. The GRADE-ADOLOPMENT processes employed in this study was in collaboration with the Section of Palliative Medicine at Aga Khan University Hospital, for the development of adult palliative care guidelines for GPs in Pakistan (Fig. 2).

### Study team

The study team consisted of the Center for Clinical Best Practices staff (all of whom have extensive experience in the formulation of management guidelines) and senior faculty and attending palliative care specialists (including the Section Head of Palliative Medicine at Aga Khan University Hospital).

### Source guideline selection

The solitary, original, “parent” clinical practice guideline that goes through the GRADE-ADOLOPMENT process for the creation of a local clinical practice guideline is termed as the source guideline. The “*National Comprehensive Cancer Network (NCCN) Guidelines ® Insights: Palliative Care, Version 2.2021* (2021)” [7] was finalized by the study team as the source guideline, due to its detailed set of recommendations, multi-disciplinary management approach, and high-quality summary of existing evidence. These guidelines incorporate pharmacological and non-pharmacological therapies in the regimen to optimize patient care according to patient and caregiver needs, with a focus on patients’ and caregivers’ mental health [7].

### Guideline review

Figure 2 illustrates the modified GRADE-ADOLOPMENT process utilized in our study, created with the collaboration of USA-GRADE working group. A Table of Recommendations (ToR) was first created by the extraction and compilation of the recommendations listed in the source guideline. Two senior attending palliative care specialists conducted independent review of the source guideline and marked each recommendation as either “*Adopt*,” “*Adapt”* or “*Exclude*.” Conflicts were resolved through consensus with the Section Head of Palliative Medicine. Recommendations marked “*Adopt*” were incorporated into the development of the local guideline with no further/minor changes, while those marked “*Exclude*” were omitted from the local guideline. Exclusions were due to the recommendation being specific to inpatient or pediatric management, or inapplicability to the local Pakistani context. Recommendations marked “*Adapt*” were recognized to require additional review and revision via the GRADEPro process before subsequent integration into the local clinical practice guidelines. This process would require best evidence review, creation of EtD tables and expert panel review as shown in the Supplementary Material. An important differentiation between our process (Fig. 2) and that described originally [15] is the absence of de novo recommendation creation, which was due to a lack of inherent necessity for further recommendations, coupled with the limitation of resources and insufficiency of research evidence available.

### GRADEPro EtD Framework

GRADEPro is a web-based application utilized for the creation, management, and sharing of research evidence summaries. Its main function is to generate EtD tables, which serve as frameworks enabling expert panel members to make healthcare recommendations or decisions based on well-summarized and balanced evidence. This evidence was to be obtained through a meticulous best evidence review process. The evaluation of recommendations in the EtD tables was to be based on a set of 12 criteria, as outlined in Supplementary Table 1. Supplementary Table 2 provides an illustrative example of an EtD. To arrive at a final consensus, an expert panel review was to be conducted. Each criterion was to be supported by evidence gathered through the best evidence review process, which provides a local context for weighing the pros and cons of the recommendation. In our study, as no recommendations warranted adaptation, we did not utilize this framework.

### Best-evidence review

To assess a recommendation across all 12 criteria, a comprehensive best-evidence review was planned. This review involved conducting separate mini-systematic reviews and examining supporting evidence. The mini-systematic review was to follow a similar protocol to a full systematic review but was to employ a specific selection criterion, such as considering the geographical region of publication, or limiting the number of searched databases. For our mini-systematic review, PubMed and Google Scholar were to be the designated databases, and a search string would be designed using keywords relevant to the recommendation under scrutiny. To maintain a local focus, only articles reporting data specific to Pakistan were to be included.

Two members of the CCBP staff were to be responsible for independently screening the titles and abstracts of the articles obtained. Only articles containing pertinent information related to Pakistan would be subjected to a thorough full-text review for final inclusion. As the source guideline was based on a systematic review process, a meticulous examination of the bibliography within the source document was essential. Additionally, two members of the CCBP staff were to be tasked with extracting relevant evidence from the finalized list of articles. In terms of supporting evidence, details concerning the cost of different investigations and treatments, as well as the availability of diagnostic and management facilities, were to be sourced from various local hospitals, healthcare facilities, and pharmacies. This information would be gathered through telephonic queries and by visiting their respective websites.

Subsequently, the CCBP team were to summarize the newly acquired evidence for each criterion in the "Research Evidence" and "Additional Considerations" columns.

### Expert panel

The completed EtD tables for each recommendation were planned to be reviewed by an expert panel comprising five senior attending palliative care specialists. Their role was to provide judgment for each criterion by selecting a response option that best aligned with their assessment. In case any expert panel member required additional evidence for a particular criterion, they were expected to communicate this to the CCBP team. The team would then make an effort to obtain the necessary information and share it with all panel members, if available.

### Referral care pathway creation

In collaboration with the Section Head of Palliative Medicine, the Center for Clinical Best Practices staff used the recommendations present in the local clinical practice guideline to create a management algorithm for primary care practitioners. The focus was on early identification and diagnosis, primary care management, and timely referral to palliative care specialists. In case of any gaps identified in the formation of the pathways, best evidence systematic review process was used to incorporate additional recommendations. Recommendations from previous authentic CPGs besides the source CPG were used. Review and approval were obtained from the experts.

### Final recommendation and referral pathway revisions & synthesis

The Center for Clinical Best Practices staff compiled the finalized recommendations into the local guideline which were then presented to the Section Head of Palliative Medicine. The referral care pathway drafted was also presented to discuss and acquire feedback after which the final clinical referral pathway was made.

### Final debriefing to identify challenges & seek solutions

Two focus group discussions were organized for the identification of challenges faced during the GRADE-ADOLOPMENT process and exploration of potential negatory solutions. They were led by a member of the Center for Clinical Best Practices team and included the Center for Clinical Best Practices staff, the Section Head of Palliative Care and expert palliative care specialists. After initially brainstorming challenges and solutions independently, participants discussed their ideas within the focus group discussions. As per consensus opinion, each challenge was classified as either a minor or major challenge. Subsequently, the Center for Clinical Best Practices team categorized the final list of specific challenges within specific themes, and their respective negatory solutions were given with them.

### Ethical considerations

The Aga Khan University's Ethics Review Committee granted a waiver of informed consent and ethics approval considering the absence of patients or other human participants. All methods adhered to the required ethical standards articulated in the *“1964 Declaration of Helsinki”* and its subsequent amendments. Important issues include the paternalistic approach to adaptation, whereby patients/public were not involved in the consensus process, with the experts serving as “proxies” for patients’ opinion. Moreover, there is a lack of representation of people of South Asian race in most of the studies that the source guideline bases its recommendations upon. Moreover, the expert consensus process in GRADE-ADOLOPMENT is inherently at risk of individual- or group-level biases.

### Timeline for palliative care CPG development in Pakistan

The methodology outlined in this study was implemented from October 2021-April 2022 as per the timeline described.

- Selection of Source Guideline: October-December 2021.
- Table of Recommendations creation: January-March 2022.
- Table of Recommendations review: March 2022.
- Finalization of Pakistani Palliative Care CPG: April 2022.

## Results

### Source guideline review

The selected source guideline [7] provided 42 comprehensive recommendations. After undergoing the GRADE-Adolopment process as shown in Fig. 2, a total of 27 were adopted without modification. No recommendations were deemed to be adapted and 15 were excluded. Supplementary Table 3 shows the details for the recommendations indicated for exclusion. The complete ToR and finalized guidelines are provided as the Supplementary Table 3 and 4 respectively.

### Referral pathways

**Fig. 3:**
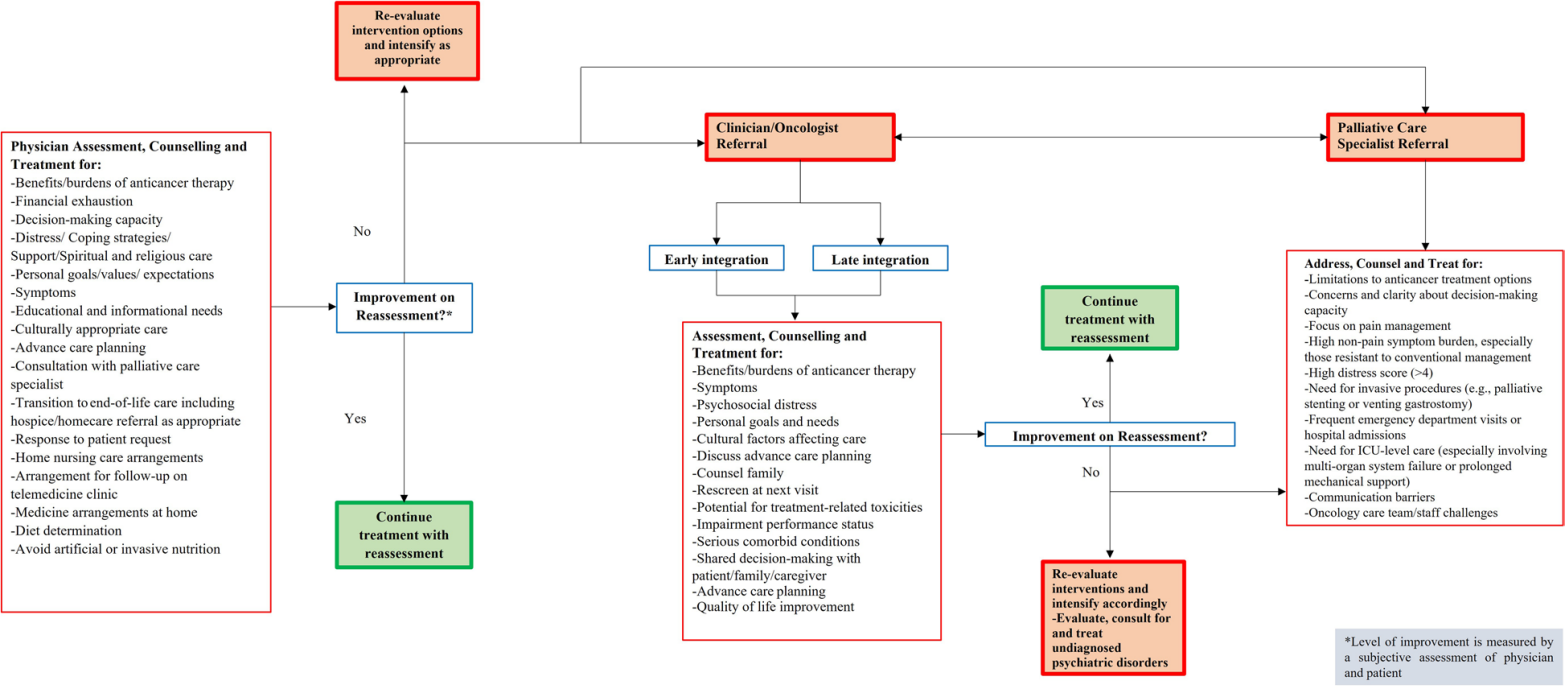
Palliative care assessment

**Fig. 4:**
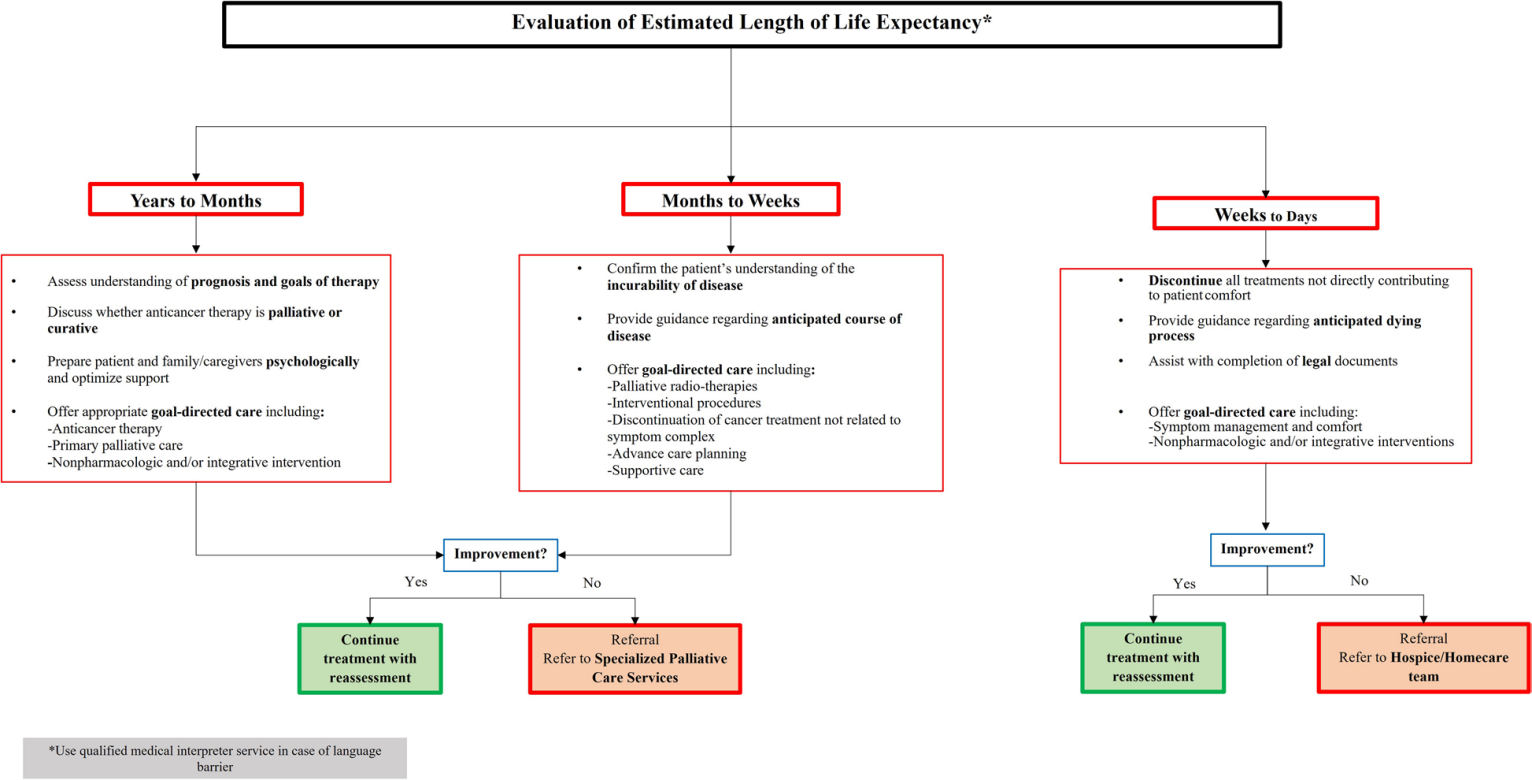
Benefits burden of anticancer treatment

**Fig. 5:**
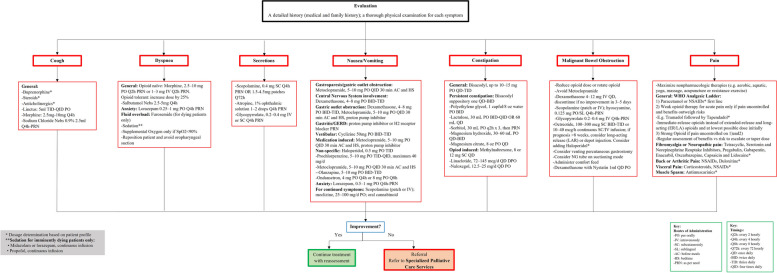
Recommended agents and dosage for all palliative patients based on symptom etiology

**Fig. 6:**
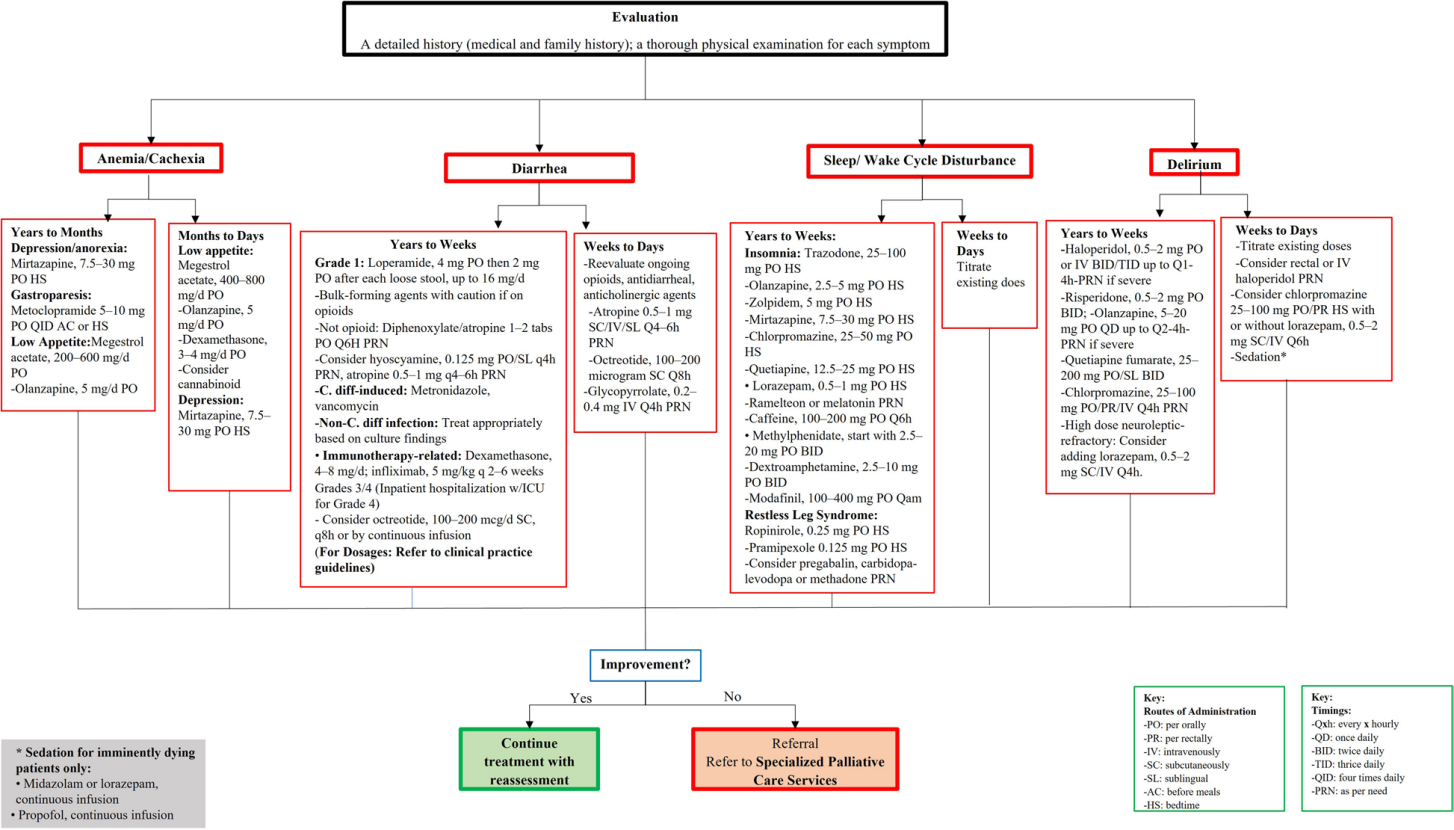
Recommended agents and dosage for palliative patients based on symptom etiology and life expectancy

Figures 3, 4, 5 and 6 show the referral pathways made from our CPG. These are algorithm-based assessment and management pathways for palliative care patients.

Table 1 shows the list of recommendations added from other CPGs to address gaps in clinical management from the guideline.

**Table 1:** Additional recommendations

	**Additional Recommendations**	**Source**
**1**	**Palliative Care Assessment, Counselling and Treatment for:**	
	• Offer spiritual and religious care • Make Home nursing care arrangements • Make Arrangement for follow-up on telemedicine clinic • Medicine arrangements at home • Diet determination • Avoid artificial or invasive nutrition	*“Advance Care Planning Guidelines for working with Asian patients and their families”* [23] *“Care of the Adult Cancer Patient at the end of Life: ESMO Clinical Practice Guidelines”* [24]
**2**	**Symptomatic Management:** **Cough: Life Expectancy: Years; Year to Months; and Months to Weeks**	
	• Buprenorphine^a^ • Steroids^a^ • Anticholinergics^a^ • Linctus: 5 ml TID-QID PO • Morphine: 2.5 mg-10 mg Q4h • Sodium Chloride Nebs 0.9% 2.5 ml Q4h-PRN	*“Palliative Care Pain & Symptom Control Guidelines for Adults”* [25] *“Scottish Palliative Care Guidelines”* [26]
**3**	**Symptomatic Management:** **Cough: Life Expectancy: Years; Year to Months; and Months to Weeks**	
	• Salbutamol Nebs 2.5-5 mg Q4h • Supplemental Oxygen only if SpO2 < 90% • Reposition patient and avoid oropharyngeal suction	*“Palliative Care Pain & Symptom Control Guidelines for Adults”* [25] *“Care of the Adult Cancer Patient at the end of Life: ESMO Clinical Practice Guidelines”* [24]
**4**	**Symptomatic Management:** **Nausea/Vomiting: Life Expectancy: Years; Year to Months; and Months to Weeks**	
	• **Vestibular:** Cyclizine 50 mg PO BID-TID	*“Palliative Care Pain & Symptom Control Guidelines for Adults”* [25]
**5**	**Symptomatic Management: Malignant Bowel Obstruction: Life Expectancy: Years; Year to Months; Months to Weeks; and Weeks to Days (dying patient)**	
	• Consider adding Haloperidol^a^ with Octreotide • Consider venting percutaneous gastrostomy • Consider NG tube on suctioning mode • Administer comfort feed • Dexamethasone with Nystatin 1 ml QD PO	*“Palliative Care Pain & Symptom Control Guidelines for Adults”* [25] *“Care of the Adult Cancer Patient at the end of Life: ESMO Clinical Practice Guidelines”* [24] *“Malignant Bowel Obstruction for Palliative Patients”* [27]
**6**	**Symptomatic Management: Pain: Life Expectancy: Years; Year to Months; Months to Weeks; and Weeks to Days (dying patient)**	
	• Maximize nonpharmacologic therapies (e.g. aerobic, aquatic, yoga, massage, acupuncture or resistance exercise) • **General: WHO Analgesic Ladder:** • 1) Paracetamol or NSAIDs^a^ first line • 2) Weak opioid therapy for acute pain only if pain uncontrolled and benefits outweigh risks • -E.g. Tramadol followed by Tapendadol^a^ • -Instead of extended-release and long-acting (ER/LA) opioids, use immediate-release opioids and at lowest possible dose initially • 3) Strong Opioid if pain uncontrolled on 1)and2) • -Regular assessment of benefits vs risk to escalate or taper dose • **Fibromyalgia or Neuropathic pain:** Tetracyclic, Serotonin and Norepinephrine Reuptake Inhibitors, Oxcarbazepine, Pregabalin, Enacarbil, Gabapentin, Lidocaine and Capsaicin^a^ • **Back or Arthritic Pain:** NSAIDs, Duloxitine^a^ • **Visceral Pain:** Corticosteroids, NSAIDs^a^ • **Muscle Spasm:** Antimuscarinics^a^	*“Palliative Care Pain & Symptom Control Guidelines for Adults”* [25] *“CDC clinical practice guideline for prescribing opioids for pain—United States, 2022”* [28] *“World Health Organization Analgesic Ladder” * [29]

^a^Dosage determination according to patient’s overall medical profile

### Challenges and solutions

Table 2 shows the challenges and solutions faced during the creation of this guideline.

**Table 2:** Challenges encountered and suggested solutions

Category of Challenge	Specific Challenge	Suggested Solution
**Resources**	Dearth of literature pertinent to our location^b^	Extensive review and utilization of available evidence
	Shortage of team members^a^	Recruiting volunteers with the required level of expertise in tasks
	Insufficient available funds^a^	Apply for grants or request external funding
**Stake Holder Support and Involvement**	Inadequate support from government, external organizations, and different stake holders^b^	-Involve all stakeholders from beginning -Emphasize on mutual interest -Send invitation to stakeholders participation in GRADE-ADOLOPMENT process
	Unavailability of general practitioner representation^b^	
	Unavailability of patient population representation^a^	
**Resistance to Change**	Difficulty in coordination between panel for availability for data review and guideline development^b^	Regular reminders with encouragement for timely task completion
	Time consuming review for data from outside our institution^a^	Employee volunteers for data extraction and search
	Experts’ doubt regarding need for local CPG, GRADE-ADOLOPMENT process credibility and feasibility^a^	Presentation to emphasize need and utility of GRADE-ADOLOPMENT process
**Methodological Limitations**	Individual level bias^b^	-Increase number of participants -Receive feedback to assess validity of recommendation reviews
	Group level bias^b^	
	Suboptimal conviction when reaching consensus among panel^a^	Maximize collection of quality literature for evidence

^a^Minor challenge; ^b^Major Challenge

## Discussion

We have aimed to create the Clinical Practice Guideline (CPG) for the management of Palliative patients using the GRADE-ADOLOPMENT process, sourced from the *“NCCN Clinical Practice Guidelines in Oncology (NCCN Guidelines®) Palliative Care, Version 2.2021”* as of December 2, 2020 [7] . Best evidence review, and expert panel input were maximized to develop the CPG and subsequent referral pathways suitable for Pakistan. From the source guidelines, we adopted all the recommendations, except for 15 which were due to repetition and reference to oncological assessment.

WHO recognizes access to palliative care as a fundamental human right [30] . Considering the escalating burden of non-communicable diseases, the need for palliative care cannot be understated anymore. WHO defines palliative care as “specialized medical care for people living with serious illness” which is incurable and/or life-threatening with drastic changes to a patient’s quality of life [31, 32] . Worldwide, a mere 14% of the population who requires palliative care is able to receive it, with most of this care concentrated in high-income countries (HICs) [33]. Concurrently, an estimated 56.8 million people are in need of this service, with the majority residing in low- and middle-income countries (LMICs) which are consequently faced with inadequate access and a high unmet need [33] . As such, it was imperative that LMICs such as Pakistan develop CPGs to implement palliative care holistically within health systems and improve access. Currently, the vast majority of CPGs that form the foundation of palliative care and are recognized have been developed by high-income Western countries. This is primarily due to their well-established practices and existing research infrastructure in palliative care. In a survey conducted by WHO, it was revealed that only 68% of countries had funding available for palliative care, however, the services were reported to reach only half the patients in need in only 40% of the countries [34] . LMICs, including Pakistan, face challenges in terms of financial resources, research capacity, and expertise, which hinder the development of local guidelines that would cater to their unique needs.

Moreover, several studies have shown a preference for at-home care or community-based care of serious patients who require palliative interventions [35] . Most people in the developed world die in an acute care setting, if not at a hospice, which contrasts with the wishes of the patients [36] . Despite the lack of adequate data, it is reasonable to assume that in a country like Pakistan, where the majority of hospital costs are through out-of-pocket financing, there is increased hesitance for inpatient hospital care in serious patients and poorly developed care options in the community which inevitably creates gaps in the continuum of care. Pakistan has a significant influence of culture and religion in our society which also requires our local CPGs to be tailored according to its principles [18] . As such, a shift to primary care pathways is paramount in improving access to quality palliative care, and cost-effectiveness but also is in line with patient preferences for community care. Therefore, the introduction of an integrated clinical guideline such as that we have proposed serves as a system-oriented strategy to enhance patient access to essential treatment, provide relief, and address the existing gap in the provision of care [37] . Thus, we utilized the GRADE-ADOLOPMENT approach to develop a CPG that incorporate the best available evidence and address the specific needs of the local context to improve Pakistan's palliative healthcare system. Not only will this guideline of care provide an objective, algorithmic approach to palliative care within general practitioners, it takes into account the socio-demographic factors of a low-resource region like Pakistan leading to the shift of specialized palliative care treatment towards primary care. [38].

Our CPG for palliative care recommends developing a plan of care that involves an interprofessional team comprising physicians, advanced practice clinicians, nurses, mental health professionals, social workers, chaplains, and other healthcare professionals. A collaborative approach aims to effectively meet the diverse requirements of patients in palliative care [39, 40] . Furthermore, the guidelines also recommend the incorporation of palliative care within general oncology care. This approach intends to improve the quality of life and ultimate survival of patients with cancer [41, 42] . Moreover, symptom and condition-specific sections in our CPG have been developed. They provide information on recommended agents, dosage adjustments based on estimated life expectancy and symptom etiology, route of administration, as well as radiography and surgical indications. Our guidelines recommend a thorough documentation of patient decisions, discussions, and agreements in the medical record. The documentation includes the use of tools such as “POLST (Physician Orders for Life-Sustaining Treatment)” or “MOLST (Medical Orders for Life-Sustaining Treatment)” to safeguard patient autonomy and guide clinical decision-making aligning with any of the patient's expressed preferences. Furthermore, the CPG also addresses mental health considerations, such as persistent complex bereavement disorder. This condition is characterized by a chronically intensified state of mourning which significantly impairs functioning, therefore, our CPG include recommendations on managing and addressing mental health challenges associated with this disorder, for holistic care. Oncology assessments and repeated recommendations were intentionally excluded from the GRADE-ADOLOPMENT process to prevent redundancy and improve clarity of the CPG.

In addition to the CPG, our team created primary care referral pathways in collaboration with local experts. These are essential as they provide a comprehensive outline for managing particular clinical conditions and aim to incorporate the most up-to-date evidence in a systematic manner [31, 43] . Such protocols reduce inter-practitioner variability of practices and improve outcomes, with reduced costs [37, 44] . The creation of algorithms, including the “Liverpool Care Pathway (LCP)” has resulted in widespread adoption and implementation of pathways in HICs such as the United States, China and Australia, showing their importance [45–47] . Similar to existing integrated care pathways, we developed primary care referral pathways in collaboration with local experts following the review and consensus on our final CPG. These pathways provide a direct link to the CPG and are an extrapolation of the existing guidelines for ease of understanding of general practitioners and improving access to palliative medicine at the level of primary care. They are user-friendly and optimized to be considerably simple and uncomplicated to comprehend for all practitioners. These pathways are objective algorithms of care for treatment decisions with the aim of improving patient outcomes in countries with low resources such as Pakistan [31, 48, 49].

We faced several challenges in the development of our CPG. With no prior existing infrastructure in palliative medicine in Pakistan outside of newly introduced services in tertiary care centers, it was difficult to establish a comprehensive care pathway which employed a multidisciplinary approach. Limited literature was available specific to our population. Moreover, it was challenging to determine the extent of care that could be provided by general practitioners at the primary care level, considering there is often suboptimal formal training in palliative medicine both in medical school and during training. It was also crucial to consider the substantial variation in the availability of resources among primary care facilities across different regions of Pakistan. Consequently, the primary emphasis was placed on initial assessment, basic management and treatment aimed at symptomatic relief.

Our study has several limitations that should be acknowledged. First, we did not incorporate input from other stakeholders involved in patient care, such as primary care physicians, other healthcare professionals, external organization experts, and policy makers. This decision was due to constraints in funding, potential conflicts of interest, and logistical difficulties. This omission may have limited the comprehensive perspective of our guidelines on palliative care in LMICs. Given that Pakistan has a significant percentage of rural areas, the feasibility of implementing our CPG in these regions may be limited due to inadequate infrastructure, resources, lack of expertise and/or availability of essential medications. Furthermore, our study defined the criteria for improvement based on the subjective judgment of physicians, introducing the potential for bias in clinical decision-making. The ToR (Terms of Reference) review process, carried out by individual experts, also presents inherent subjectivity and the possibility of bias when determining whether to “adopt”, “adapt”, or “create” recommendations. These limitations underscore the practical challenges and potential biases that arise when applying the optimal GRADE-ADOLOPMENT process, particularly in LMICs such as Pakistan. Nonetheless, efforts can be made to address and mitigate these limitations in future endeavours. While our CPG primarily focuses on management of palliative care for cancer patients, the burden of non-malignancy patients is also significant, possibly limiting the generalizability of our guideline. However, the framework laid can be used to emulate management for other non-malignancy related palliative-care situations for similar effective care.

## Conclusion

Provision of palliative care is an overlooked aspect in society. Through the GRADE-ADOLOPMENT process, we have created the first CPG for its provision in Pakistan, considering the local considerations. We have also created easy to comprehend referral pathways from our CPG. Dispersion and usage of these will help improve the patient outcomes and refine the facilitation of palliative care.

### Supplementary Information

**Supplementary Material 1.**

## Data Availability

All data acquired for this research is included in the manuscript.
