# Supplementary material for: Development of palliative care clinical practice guidelines and referral care pathways for primary care practitioners in Pakistan
Source: BMC Palliat Care. 2024 May 1;23:112. doi: 10.1186/s12904-024-01438-y (PMC11061908; doi:10.1186/s12904-024-01438-y)
Supplement: Supplementary file 1 — Supplementary Material 1. [file 12904_2024_1438_MOESM1_ESM.docx]

**Supplementary Material**

1. *Supplementary Table 1: Criteria evaluated in the Evidence to Decision (EtD) table*
2. *Supplementary Table 2: Dummy Evidence to Decision (EtD) table*
3. *Supplementary Table 3: Table of Recommendations for the Intervention*
4. *Supplementary Table 4: Finalized Guidelines*

| **Supplementary Table 1: Criteria evaluated in the Evidence-to-Decision tables** | | |
| --- | --- | --- |
| **Criteria** | **Description** | **Interpretation** |
| **Problem** | The magnitude of a problem, as measured by its prevalence and severity in a local context | The more serious or urgent a problem is, the more likely that the option that better addresses the problem receives a strong recommendation. |
| **Desirable Effects** | The magnitude of desirable effects is judged by considering the importance of the outcome and the size of the desirable effects (likelihood of experiencing a benefit or degree of benefits an individual experiences). | An option with greater desirable effects is more likely to gain a strong recommendation. |
| **Undesirable Effects** | The magnitude of undesirable effects is similarly judged by considering the importance of the outcome and the size of the undesirable effects. | An option with fewer undesirable effects is more likely to gain a strong recommendation. |
| **Certainty of Evidence** | Determined by likelihood that the research provides valid evidence regarding the effect of the option on all critical outcomes. | Evidence with higher certainty lends to a strong recommendation. |
| **Values** | Magnitude of value is judged by the variability or uncertainty of weightage placed upon the outcome by individuals. | Less variability/uncertainty of value leads to a strong recommendation. |
| **Balance of Effects** | The balance of effects is judged by considering the value individuals place upon the main outcomes, the degree of desirable and undesirable effects, and the certainty of those estimates. | The overall balance of effects can be judged as either favoring the intervention or comparison. |
| **Resources Required** | An estimate of the cost of the difference in resource use between the intervention and comparison. | An option with large savings is more likely to receive a strong recommendation. |
| **Certainty of Evidence of Required Resources** | It is determined by the likelihood that the research provides valid evidence of cost differences between the intervention and comparison. | Evidence with higher certainty lends to a strong recommendation. |
| **Cost-Effectiveness** | Determines cost-effectiveness by considering uncertainty about or variability in costs or net benefit, sensitivity analyses, and the reliability and applicability of the economic evaluation. | An option that is more cost-effective is more likely to receive a strong recommendation |
| **Equity** | Likelihood of differences in the relative effectiveness of the intervention for disadvantaged subgroups that influence the absolute effectiveness of the intervention. | An option with a greater likelihood to favor equity is more likely to receive a strong recommendation. |
| **Acceptability** | Likelihood of key stakeholders to accept the distribution of benefits, harms, costs, and ethical concerns associated with the intervention, over an extended period. | An option more acceptable to most stakeholders is more likely to receive a strong recommendation. |
| **Feasibility** | Practicality of sustained use of the intervention. | An option more feasible to most stakeholders is more likely to receive a strong recommendation. |

| **Supplementary Table 2: Evidence to Decision table unfilled sample** | | | |
| --- | --- | --- | --- |
| **Question**: Should *Intervention/Suggested Change* be favored over *Comparison/Current Standard of Practice*? | | | |
| **Criteria** | **Research Evidence** | **Additional Considerations** | **Judgment** |
| **Problem:** Is the problem a priority? |  |  | - No - Probably No - Probably Yes - Yes - Varies - Don’t Know |
| **Desirable Effects:** How substantial are the desirable anticipated effects? |  |  | - Trivial - Small - Moderate - Large - Varies - Don’t Know |
| **Undesirable Effects**: How substantial are the undesirable anticipated effects? |  |  | - Large - Moderate - Small - Trivial - Varies - Don’t Know |
| **Certainty of Evidence**: What is the overall certainty of the evidence of effects? |  |  | - Very Low - Low - Moderate - High - No Included Studies |
| **Value**: Is there important uncertainty about or variability in how much people value the main outcomes? |  |  | - Important Uncertainty or Variability - Possible Uncertainty or Variability - Probably No Important Uncertainty or Variability - No Important Variability or Uncertainty |
| **Balance of Effects**: Does the balance between desirable and undesirable effects favor the intervention or the comparison? |  |  | - Favors Comparison - Probably Favors the Comparison - Does Not Favor Either the Intervention or Comparison - Probably Favors the Intervention - Favors Intervention - Varies - Don’t Know |
| **Resources Required**: How large are the resource requirements (costs)? |  |  | - Large Costs - Moderate Costs - Negligible Costs or Savings - Moderate Savings - Large Savings - Varies - Don’t Know |
| **Certainty of Evidence of Required Resources**: What is the certainty of the evidence of resource requirements (costs)? |  |  | - Very Low - Low - Moderate - High - No Included Studies |
| **Cost-Effectiveness**: Does the cost-effectiveness of the intervention favor the intervention or the comparison? |  |  | - Favors Comparison - Probably Favors the Comparison - Does Not Favor Either the Intervention or Comparison - Probably Favors the Intervention - Favors Intervention - Varies - No Included Studies |
| **Equity**: What would be the impact on health equity? |  |  | - Reduced - Probably Reduced - Probably No Impact - Probably Increased - Increased - Varies - Don’t Know |
| **Acceptability**: Is the intervention acceptable to key stakeholders? |  |  | - No - Probably No - Probably Yes - Yes - Varies - Don’t Know |
| **Feasibility**: Is the intervention feasible to implement? |  |  | - No - Probably No - Probably Yes - Yes - Varies - Don’t Know |
| **Overall Recommendations**   - Strong Recommendation Against the Intervention - Conditional Recommendation Against the Intervention - Conditional Recommendation for Either the Intervention or the Comparison - Conditional Recommendation for the Intervention - Strong Recommendation for the Intervention | | | |

**Supplementary Table 3. Table of Recommendations for the Intervention**

| S.no | Recommendations | To adopt | To Adapt | To Exclude |
| --- | --- | --- | --- | --- |
| 1 | Patients with one or more positive indicators may benefit from a care plan developed by an interprofessional team of physicians, nurses, social workers, mental health professionals, chaplains, advanced practice clinicians, and other health care professionals. | X |  |  |
| 2 | Oncologists should integrate palliative care into general oncology care. Early consultation/collaboration with a palliative care specialist/hospice team should be considered to improve quality of life and survival. | X |  |  |
| 3 | Patients with one or more positive indicators may benefit from a care plan developed by an interprofessional team of physicians, nurses, social workers, mental health professionals, chaplains, advanced practice clinicians, and other health care professionals. |  |  | X  Repeated/redundant |
| 4 | Oncologists should integrate palliative care into general oncology care. Early consultation/collaboration with a palliative care specialist/hospice team should be considered to improve quality of life and survival. |  |  | X  Repeated/redundant |
| 5 | For an approach to assess for the risk of serious complications in patients with neutropenic fever [See FEV-D in the NCCN Guidelines for Prevention and Treatment of Cancer-Related Infections.](https://www.nccn.org/professionals/physician_gls/pdf/infections.pdf) (1) |  |  | X  Oncology Assessments are excluded from the Manual |
| 6 | For an approach to decision-making in older adults and geriatric screening tools, [see the NCCN Guidelines for Older Adult Oncology](https://www.nccn.org/professionals/physician_gls/pdf/senior.pdf). (1) |  |  | X  Oncology Assessments are excluded from the Manual |
| 7 | For an approach to assess for the risk of serious complications in patients with neutropenic fever.  [See FEV-D in the NCCN Guidelines for Prevention and Treatment of Cancer-](https://www.nccn.org/professionals/physician_gls/pdf/infections.pdf) [Related Infections.](https://www.nccn.org/professionals/physician_gls/pdf/infections.pdf) (1) |  |  | X Repeated |
| 8 | For an approach to decision-making in older adults and geriatric screening tools  [see the NCCN Guidelines for Older Adult Oncology](https://www.nccn.org/professionals/physician_gls/pdf/senior.pdf). (1) |  |  | X Repeated |
| 9 | Look for opportunities to use single agents to treat multiple symptoms | X |  |  |
| 10 | For an approach to assess for the risk of serious complications in patients with neutropenic fever  [See FEV-D in the NCCN Guidelines for Prevention and Treatment of Cancer-Related Infections. (1)](https://www.nccn.org/professionals/physician_gls/pdf/infections.pdf) |  |  | X Repeated |
| 11 | Look for opportunities to use single agents to treat multiple symptoms. |  |  | X Repeated |
| 12 | Patients with one or more positive indicators may benefit from a care plan developed by an interprofessional team of physicians, nurses, social workers, mental health professionals, chaplains, advanced practice clinicians, and other health care professionals |  |  | X Repeated |
| 13 | Oncologists should integrate palliative care into general oncology care. Early consultation/collaboration with a palliative care sspecialist/hospice team should be considered to improve quality of life and survival. |  |  | X Repeated |
| 14 | Persistent complex bereavement disorder is a chronic heightened state of mourning that significantly impairs functioning | X |  |  |
| 15 | For specific recommendations for medical management of symptoms.   \|  \| Condition \| Recommended Agents and Dosage by Estimated Life Expectancy and Symptom Etiology \| \| --- \| --- \| --- \| \| 15.a \| Dyspnea \| Life Expectancy: Years; Year to Months; and Months to Weeks   - General: Morphine, 2.5–10 mg PO q2h PRN or 1–3 mg IV q2h PRN for opioid naïve, increase dose by 25% for opioid tolerant   For acute progressive dyspnea, or for patients who are not opioid naïve, more aggressive titration may be required   - Anxiety: Lorazepam, 0.25–1 mg PO q4h PRN for benzodiazepine naïve \| \| 15.b \| Dyspnea \| Life Expectancy: Weeks to Days (dying patient)   - General: Morphine, 2.5–10 mg PO q2h PRN or 1–3 mg IV q2h PRN if opioid naïve, increase dose by 25% for opioid tolerant - For acute progressive dyspnea, or for patients who are not opioid naïve, more aggressive titration may be required. - Anxiety: Lorazepam, 0.25–1 mg PO q4h PRN if benzodiazepine nïve - Fluid overload: Furosemide \| \| 15.c \| Secretions \| - Excessive secretions: Scopolamine, 0.4 mg SC q4h PRN/1.5 mg patches, 1–3 patches q72h OR atropine, 1% ophthalmic solution 1–2 drops SL q4h PRN OR glycopyrrolate, 0.2–0.4 mg IV or SC q4h PRN \| \| 15.d \| Anorexia/Cachexia \| Life Expectancy: Years; Year to Months   - Depression/anorexia: Mirtazapine, 7.5–30 mg PO QHS - Gastroparesis (early satiety): Metoclopramide 5–10 mg PO QID 30 min before meals and at bedtime - Low/no appetite: Megestrol acetate, 200–600 mg/d PO OR olanzapine, 5 mg/d PO \| \| 15.e \| Anorexia/ Cachexia \| Life Expectancy: Months to Weeks; Weeks to Days (dying patient)   - Low/no appetite: Megestrol acetate, 400–800 mg/d d PO OR olanzapine, 5 mg/d PO OR dexamethasone, 3–4 mg/d PO OR consider cannabinoid - Depression: Mirtazapine, 7.5–30 mg PO QHS \| \| 15.d \| Nausea and Vomiting (NV)- Initial Treatment \| Life Expectancy: Years; Year to Months; Months to Weeks; and Weeks to Days (dying patient)  •See NCCN Guidelines for Antiemesis for chemotherapy/radiation-induced NV  •Gastroparesis: Metoclopramide, 5–10 mg PO QID 30 min before meals and at bedtime  •CNS involvement: Dexamethasone, 4–8 mg PO BID-TID  •Gastric outlet obstructions: Dexamethasone, 4–8 mg/d PO; proton pump inhibitor; metoclopramide, 5–10 mg PO QID 30 min before  meals and at bedtime  •Gastritis/GERD: Proton pump inhibitor OR H2 blocker  •Medication-induced gastropathy: Proton pump inhibitor OR metoclopramide, 5–10 mg PO QID 30 min before meals and at bedtime  Nonspecific NV  •Dopamine receptor antagonists or 5-HT3 receptor antagonists  ◗Haloperidol, 0.5 mg PO TID OR metoclopramide, 5–10 mg PO QID 30 min before meals and at bedtime OR prochlorperazine, 5–10 mg PO 3–4 times/d, maximum 40 mg/d OR olanzapine, 5–10 mg PO 2–3 times/d OR ondansetron, 4 mg PO q4h or 8 mg PO q8h  •Contributing anxiety: Lorazepam, 0.5–1 mg PO q4h PRN  •Vertiginous component: Anticholinergic AND/OR antihistamine \| \| 15.e \| Nausea and Vomiting (NV)- Initial Treatment \| Life Expectancy: Years; Year to Months; Months to Weeks; and Weeks to Days (dying patient)  •Consider appropriate route of administration  ◗1) Prescribe oral, sublingual, or rectal agent and titrate to maximum benefit  ◗2) If NV persists, provide PRN, scheduled, or continuous parenteral infusion as necessary  ◗3) Consider subcutaneous administration as an alternative  •Titrate to maximum benefit and tolerance: olanzapine, prochlorperazine, haloperidol, or metoclopramide  •For continued NV, consider additional agents:  ◗Dexamethasone, 4–8 mg/d PO; ondansetron, 4–8 mg PO every 6 h; scopolamine (patch or IV); meclizine, 25–100 mg/d PO; oral  cannabinoid \| \| 15.f \| Constipation \| Life Expectancy: Years; Year to Months; Months to Weeks; and Weeks to Days (dying patient)  •Prophylaxis: Titrate the senna and add polyethylene glycol, recommend starting with polyethylene glycol if the patient is not on opioids and can tolerate the volume of liquid  •General: Add bisacodyl, titrate to 10–15 mg PO daily-TID with a goal of 1 non-forced bowel movement (BM) every 1–2 days  •Persistent constipation: bisacodyl suppository, one rectally daily-BID; polyethylene glycol, 1 capful/8 oz water PO BID; lactulose, 30 mL PO BID-QID OR 60 mL daily; sorbitol, 30 mL PO q2h x 3, then PRN; magnesium hydroxide, 30–60 mL PO daily-BID; or magnesium citrate, 8 oz PO daily  •Opioid-induced constipation: Consider methylnaltrexone, 8 or 12 mg/dose SC, no more than once a day; linaclotide, 72–145 mcg/d PO; naloxegol, 12.5–25 mg/d PO  ◗Not for post-op ileus and mechanical bowel obstruction \| \| 15.g \| Diarrhea \| Life Expectancy: Years; Year to Months; and Months to Weeks  Grade 1  •General: Loperamide, 4 mg PO x 1 then 2 mg PO after each loose stool, up to 16 mg/d  •Consider addition of bulk-forming agents with caution in patients on opioids  •If not on opioids: Diphenoxylate/atropine 1–2 tabs PO q6h PRN, maximum 8 tabs/d Grade 2  •Initiate/continue loperamide, 4 mg PO x 1 then 2 mg PO after each loose stool, up to 16 mg/d  •If not on opioids: Diphenoxylate/atropine 1–2 tabs PO q6h PRN, maximum 8 tabs/d  •Consider hyoscyamine, 0.125 mg PO/ODT/SL q4h PRN, max: 1.5 mg/d; atropine 0.5–1 mg SC/IM/IV/SL q 4–6h PRN  •C. diff-induced: Metronidazole, 500 mg PO/IV QID x 10–14 days; vancomycin, 125–500 mg PO QID x 10–14 days  •Non-C. diff infection: Treat appropriately based on culture findings  •Immunotherapy-related: Dexamethasone, 4–8 mg/d; infliximab, 5 mg/kg q 2–6 weeks  Grades 3/4 (Inpatient hospitalization w/ICU for Grade 4)  •Initiate/continue loperamide, 4 mg PO x 1 then 2 mg PO after each loose stool, up to 16 mg/d  •If not on opioids: Diphenoxylate/atropine, 1–2 tabs PO q6h PRN, maximum 8 tabs/d  •Consider hyoscyamine, 0.125 mg PO/ODT/SL q4h PRN, max: 1.5 mg/d; atropine 0.5–1 mg SC/IM/IV/SL q 4–6 h PRN  •Consider octreotide, 100–200 mcg/d SC, q8h or by continuous infusion \| \| 15.h \| Diarrhea \| Life Expectancy: Weeks to Days (dying patient)  •Reevaluate ongoing antidiarrheal, anticholinergic agents  •Initiate or increase dose of around-the-clock opioid  •Atropine 0.5–1 mg SC/IV/SL q 4–6h PRN  •Consider octreotide, 100–200 microgram SC q8h  •Consider glycopyrrolate, 0.2–0.4 mg IV q4h PRN \| \| 15.i \| Malignant Bowel Obstruction \| Life Expectancy: Years; Year to Months; Months to Weeks; and Weeks to Days (dying patient)  •Reduce opioid dose or rotate opioid  •Metoclopramide, 5–10 mg PO QID 30 min before meals and at bedtime; avoid in the setting of complete obstruction  •Dexamethasone, 4–12 mg IV daily, discontinue if no improvement in 3–5 days  •Scopolamine (patch or IV); hyoscyamine, 0.125 mg PO/ODT/SL q4h PRN; glycopyrrolate, 0.2–0.4 mg IV q4h PRN  •Octreotide, 100–300 mcg SC BID-TID or 10–40 mcg/h continuous SC/IV infusion; if prognosis >8 weeks, consider long-acting release  (LAR) or depot injection \| \| 15.j \| Sleep/Wake Disturbance \| Life Expectancy: Years; Year to Months; and Months to Weeks  Insomnia  •Trazodone, 25–100 mg PO at bedtime  •Olanzapine, 2.5–5 mg PO at bedtime  •Zolpidem, 5 mg PO at bedtime  •Mirtazapine, 7.5–30 mg PO at bedtime  •Chlorpromazine, 25–50 mg PO at bedtime  •Quetiapine, 12.5–25 mg PO at bedtime  •Lorazepam, 0.5–1 mg PO at bedtime  •For phase shift disorder consider ramelteon (8 mg PO 30 min before bedtime) or melatonin (30 min before bedtime; dosage may vary  by formulation) Daytime Sedation  •Caffeine, 100–200 mg PO q 6 h, last dose 4 PM  •Methylphenidate, start with 2.5–20 mg PO BID, second dose no later than 6 h before bedtime  •Dextroamphetamine, 2.5–10 mg PO BID, second dose no later than 12 h before bedtime  •Modafinil, 100–400 mg PO each morning  Restless Legs Syndrome (RLS)  •Ropinirole, 0.25 mg PO 1–3 h before bedtime  •Pramipexole, starting dose 0.125 mg PO at bedtime, may require titration  •May also consider pregabalin, carbidopa-levodopa, or low-dose methadone with dopamine agonist; however, all of these medications are off-label for RLS \| \| 15.k \| Sleep/Wake Disturbance \| Life expectancy: Weeks to Days (dying patient)  •Titrate dose of existing pharmacotherapy \| \| 15.l \| Delirium \| Life Expectancy: Years; Year to Months; and Months to Weeks  Mild/Moderate Delirium  •Haloperidol, 0.5–2 mg PO BID/TID  •Alternatives: risperidone, 0.5–2 mg PO BID; olanzapine, 5–20 mg PO daily; or quetiapine fumarate, 25–200 mg PO/SL BID  Severe Delirium (agitation)  •Haloperidol, 0.5–2 mg IV q1–4h PRN  •Alternatives: olanzapine, 2.5–7.5 mg PO/SL q2–4h PRN (maximum = 30 mg/d); chlorpromazine, 25–100 mg PO/PR/IV q4h PRN  for bedbound patients  •4High-dose neuroleptic-refractory: Consider adding lorazepam, 0.5–2 mg SC/IV q4h \| \| 15.m \| Delirium \| Life Expectancy: Weeks to Days (dying patient)  •Upward titrate haloperidol, risperidone, olanzapine  •High-dose neuroleptic-refractory: Upward titrate lorazepam  •Consider rectal or IV haloperidol  •Consider chlorpromazine, 25–100 mg PO/PR at bedtime with or without lorazepam, 0.5–2 mg SC/IV q6h \| \| 15.n \| Palliative Sedation \| Imminently dying patient  •Midazolam or lorazepam, continuous infusion  •Propofol, continuous infusion \| |  |  |  |
|  |  | X  X  X  X  X  X  X  X  X  X  X  X  X  X  X  X  X |  |  |
| 16 | For acute progressive dyspnea or for patients who are not opioid naive, more aggressive titration may be required | X |  |  |
| 17 | The addition of benzodiazepines to opioids can increase the risk of respiratory depression | X |  |  |
| 18 | An around-the-clock dosing schedule may provide the most consistent benefit to the patient. |  |  |  |
| 19 | Continuous intravenous or subcutaneous infusions of different antiemetics may be necessary for the management of intractable NV | X |  |  |
| 20 | Use suppository and enema with caution in patients receiving chemotherapy due to risk of cytopenia. |  |  | X  Oncology Assessments are excluded from the Manual |
| 21 | Plain film radiography may be helpful in confirming the clinical diagnosis of bowel obstruction. Consider a CT scan if surgical intervention is contemplated, as it is more sensitive and may help identify the cause of obstruction. | X |  |  |
| 21 | Most malignant bowel obstructions are partial, allowing time to discuss appropriate intervention with the patient/family/caregiver | X |  |  |
| 22 | Persistent complex bereavement disorder is a chronic heightened state of mourning that significantly impairs functioning. |  |  | X Repeated |
| 23 | Persistent complex bereavement disorder is a chronic heightened state of mourning that significantly impairs functioning. |  |  |  |
| 24 | Patient's values and preferences and any decisions should be documented in the medical record, including MOLST/POLST (Medical Orders for Life-Sustaining Treatment or Physician Orders for Life-Sustaining Treatment). | X |  |  |
| 25 | Patient's values and preferences and any decisions should be documented in the medical record, including MOLST/POLST |  |  | X Repeated |
| 26 | Persistent complex bereavement disorder is a chronic heightened state of mourning that significantly impairs functioning |  |  | X Repeated |

**Supplementary Table 4. Finalized Guidelines for Palliative Care in Pakistan**

|  | **Assessment** |
| --- | --- |
|  | Assessment of patients to be based on   - Benefits/burdens of anticancer therapy - Financial toxicity - Decision-making capacity - Coping strategies - Personal goals/values/ expectations - Symptoms - Psychosocial or spiritual distress - Educational and informational needs - Cultural factors affecting care - Criteria for consultation with palliative care specialist - Advance care planning - Psychosocial and spiritual support - Culturally appropriate care - Resource management/ social support - Consultation with palliative care specialist - Transition to end-of-life care including hospice/homecare referral as appropriate - Response to request to withdraw or withhold life- sustaining treatment - Response to requests for hastened death (physician assisted dying) |
|  | **Palliative Care Interventions** |
|  | - Appropriate treatment of comorbid physical and psychosocial conditions - Coordination of care with other health care providers - Promote adaptive coping - Symptom management - Advance care planning - Psychosocial and spiritual support - Culturally appropriate care - Resource management/ social support - Consultation with palliative care specialist - Transition to end-of-life care including hospice/homecare referral as appropriate - Response to request to withdraw or withhold life- sustaining treatment - Care of imminently dying hospitalized patient - Palliative sedation |
|  | **Reassessment** |
| Refer  to  Specialist | **Acceptable outcomes:**  - Adequate symptom management - Reduction of patient/family/ caregiver distress - Acceptable sense of control - Decision-making capacity - Decreased caregiver burden - Strengthened relationships - Optimized quality of life - Personal growth and enhanced meaning   **If unacceptable**:   - Re-evaluate intervention options and intensify as possible - Consult with other clinicians and refer to specialist if available |
|  | Appropriate treatment of comorbid physical and psychosocial conditions   - Coordination of care with other health care providers - Promote adaptive coping - Symptom management |
|  | **Assessment by Oncology Team** |
| Refer  to  Specialist | - Benefits/burdens of anticancer therapy  Symptoms  - Psychosocial distress - Personal goals/values/ expectations - Educational and informational needs - Cultural factors affecting care - Criteria for consultation with a palliative care specialist - Inform the patient/family/caregiver about palliative care services: - Anticipate symptoms and discuss preventive measures - Discuss advance care planning - Rescreen at next visit - Natural history of specific tumor - Potential for response to further treatment - Potential for treatment-related toxicities - Patient’s understanding of disease prognosis - Hopes for and understanding of anticancer therapy - Impairment of vital organs - Performance status - Serious comorbid conditions - Shared decision-making with patient/family/caregiver - Advance care planning - Hopes for and understanding of anticancer therapy - Quality of life - Symptom management - Psychiatric depression - Psychosocial distress. - Education and informational needs - Cultural factors affecting care |
|  | **Oncology Team Interventions** |
| Refer  to  Specialist | **Collaborate with palliative care specialist/team** |
|  | **Collaborate with other health care professionals treating the patient** |
|  | - Consider additional referrals: - Mental health and social services - Spiritual care - Health care interpreters - Others - Mobilize community support: - Religious - School - Community agencies - Expedite referral to hospice/homecare services when appropriate |
|  | **Reassessment** |
|  | **Acceptable outcomes:**   - Patient satisfied with response to anti-cancer therapy. - Adequate symptom management - Reduction of patient/family/caregiver distress - Acceptable sense of control - Decreased caregiver burden - Strengthened relationships - Optimized quality of life - Personal growth and enhanced meaning - Advance care planning in progress   Ongoing re-evaluation and communication between the patient and health care team |
|  | **If unacceptable**:   - Re-evaluate intervention options and intensify as possible - Evaluate and treat undiagnosed psychiatric disorders, and substance use disorders - Consult with the following: - Mental health professional - Addiction specialist - Promote adaptive coping |
|  | Patients with **one or more positive indicators*** may benefit from a care plan developed by an interprofessional team of physicians, nurses, social workers, mental health professionals, spiritual healers, advanced practice clinicians, and other health care professionals. |
| Refer  to  Specialist | Oncologists should **integrate palliative care into general oncology** care. Early consultation/collaboration with a palliative care specialist/hospice/homecare team should be considered to improve quality of life and survival. |
|  | Look for opportunities to use single agents to treat multiple symptoms |
|  | **Palliative Care Specialist Intervention** |
|  | Limited anticancer treatment options due to:   - Limited access to anticancer treatment - Advanced disease process - Multiple and/or severe comorbid conditions - Rapidly progressive functional decline or persistently poor performance status   • Concerns about decision-making capacity  • Need for clarification of goals of care  • Resistance to engage in advance care planning  • High risk of poor pain management or pain that remains resistant to conventional interventions, eg:   - Neuropathic pain - Incident or breakthrough pain - Pain with severe associated psychosocial and/or family distress - Rapid escalation of opioid dose - History of multiple adverse reactions to pain and symptom management interventions - History of diagnosis/suspicion of substance use disorder - High non-pain symptom burden, especially those resistant to conventional management - High distress score (>4) - Need for invasive procedures (eg, palliative stenting or venting gastrostomy) - Frequent emergency department visits or hospital admissions - Need for ICU-level care (especially involving multi-organ system failure or prolonged mechanical support)   Communication barriers:   - Language - Literacy - Culture - Physical barriers - Cognitive impairment   Complex patient/family/caregiver circumstances  Oncology care team/staff challenges |
|  | **Benefits burdens of anti-cancer therapy** |
|  | **Estimated Life Expectancy: Years to months** |
|  | - Assess **understanding** of prognosis and goals of therapy - Discuss whether anticancer therapy is **palliative or curative** - Prepare patient **psychologically** for possible disease progression or recurrence - Offer **goal-directed supportive care**, including referral to specialized palliative care services, if indicated - Provide appropriate anticancer therapy that is **aligned** with stated patient goals and priorities - Provide **primary palliative care**, including anti-cancer treatment and disease-related symptom management and encouragement of advance care planning - Optimize **psychosocial support** for patient and family/caregivers - Consider nonpharmacologic and/or integrative interventions (eg, massage, art or music therapy) |
|  | **Estimated Life Expectancy: Months to weeks** |
|  | - Confirm the patient's **understanding** of incurability of disease   - **Redirect goals and hopes** to those that are achievable based on likely prognosis and life expectancy   - Provide **guidance** regarding anticipated course of disease   - consider palliative RT:   - Assess for **appropriateness** of palliative RT therapies orinterventional procedures   - Consider **discontinuation** of cancer treatment not directly addressing a symptom complex   - Encourage **advance care planning**, if not already accomplished   - Offer **goal-directed supportive care**, including referral to specialized palliative care services or hospice/homecare. |
|  | **Estimated Life Expectancy: Weeks to days (dying patient)- end of life care** |
|  | - - **Discontinue** all treatments not directly contributing to patient comfort   - **Intensify palliative care** in preparation for death   - Provide guidance regarding **anticipated dying process**   - Focus on **symptom management** and comfort   - **Refer** to hospice/homecare team   - Assist with completion of **legal** documents, if appropriate   - Consider **nonpharmacologic and/or integrative interventions** (eg, heat or ice, positioning or support) |
|  | **Recommended Agents and Dosage by Estimated Life Expectancy and Symptom Etiology** |
|  | **Dyspnea: Life Expectancy: Years; Year to Months; and Months to Weeks** |
|  | - **General:** Morphine, 2.5–10 mg PO q2h PRN or 1–3 mg IV q2h PRN for opioid naïve, increase dose by 25% for opioid tolerant - For acute progressive dyspnea, or for patients who are not opioid naïve, more aggressive titration may be required   **Anxiety:** Lorazepam, 0.25–1 mg PO q4h PRN for benzodiazepine naïve |
|  | **Dyspnea Life Expectancy: Weeks to Days (dying patient)** |
|  | - **General:** Morphine, 2.5–10 mg PO q2h PRN or 1–3 mg IV q2h PRN if opioid naïve, increase dose by 25% for opioid tolerant - For acute progressive dyspnea, or for patients who are not opioid naïve, more aggressive titration may be required. - **Anxiety:** Lorazepam, 0.25–1 mg PO q4h PRN if benzodiazepine naïve - **Fluid overload:** Furosemide |
|  | **Secretions** |
|  | **Excessive secretions**: Scopolamine, 0.4 mg SC q4h PRN/1.5 mg patches, 1–3 patches q72h OR atropine, 1% ophthalmic solution 1–2 drops SL q4h PRN OR glycopyrrolate, 0.2–0.4 mg IV or SC q4h PRN |
|  | **Anorexia/ Cachexia Life Expectancy: Years; Year to Months** |
|  | - **Depression/anorexia**: Mirtazapine, 7.5–30 mg PO QHS - **Gastroparesis (early satiety):** Metoclopramide 5–10 mg PO QID 30 min before meals and at **bedtime** - **Low/no appetite:** Megestrol acetate, 200–600 mg/d PO OR olanzapine, 5 mg/d PO |
|  | **Anorexia/ Cachexia Life Expectancy: Months to Weeks; Weeks to Days (dying patient)** |
|  | - **Low/no appetite:** Megestrol acetate, 400–800 mg/d d PO OR olanzapine, 5 mg/d PO OR dexamethasone, 3–4 mg/d PO OR consider cannabinoid - **Depression:** Mirtazapine, 7.5–30 mg PO QHS |
|  | **Nausea and Vomiting (NV)- Initial Treatment Life Expectancy: Years; Year to Months; Months to Weeks; and Weeks to Days (dying patient)** |
|  | - See NCCN Guidelines for Antiemesis for chemotherapy/radiation-induced NV   •**Gastroparesis:** Metoclopramide, 5–10 mg PO QID 30 min before meals and at bedtime  •**CNS involvement:** Dexamethasone, 4–8 mg PO BID-TID  •**Gastric outlet obstructions:** Dexamethasone, 4–8 mg/d PO; proton pump inhibitor; metoclopramide, 5–10 mg PO QID 30 min before  meals and at bedtime  •**Gastritis/GERD:** Proton pump inhibitor OR H2 blocker  •**Medication-induced gastropathy:** Proton pump inhibitor OR metoclopramide, 5–10 mg PO QID 30 min before meals and at bedtime  **Nonspecific NV**  •Dopamine receptor antagonists or 5-HT3 receptor antagonists  Haloperidol, 0.5 mg PO TID OR metoclopramide, 5–10 mg PO QID 30 min before meals and at bedtime OR prochlorperazine, 5–10 mg PO 3–4 times/d, maximum 40 mg/d OR olanzapine, 5–10 mg PO 2–3 times/d OR ondansetron, 4 mg PO q4h or 8 mg PO q8h  •**Contributing anxiety:** Lorazepam, 0.5–1 mg PO q4h PRN  •**Vertiginous component:** Anticholinergic AND/OR antihistamine |
|  | **Nausea and Vomiting (NV)- Initial Treatment Life Expectancy: Years; Year to Months; Months to Weeks; and Weeks to Days (dying patient)** |
|  | • Consider **appropriate route of administration**  1) Prescribe oral, sublingual, or rectal agent and titrate to maximum benefit  2) If NV persists, provide PRN, scheduled, or continuous parenteral infusion as necessary  3) Consider subcutaneous administration as an alternative  •Titrate to maximum benefit and tolerance: olanzapine, prochlorperazine, haloperidol, or metoclopramide  •For continued NV, consider additional agents:  Dexamethasone, 4–8 mg/d PO; ondansetron, 4–8 mg PO every 6 h; scopolamine (patch or IV); meclizine, 25–100 mg/d PO; oral  cannabinoid |
|  | **Constipation Life Expectancy: Years; Year to Months; Months to Weeks; and Weeks to Days (dying patient)** |
|  | • **Prophylaxis:** Titrate the senna and add polyethylene glycol, recommend starting with polyethylene glycol if the patient is not on opioids and can tolerate the volume of liquid  • **General:** Add bisacodyl, titrate to 10–15 mg PO daily-TID with a goal of 1 non-forced bowel movement (BM) every 1–2 days  • **Persistent constipation:** bisacodyl suppository, one rectally daily-BID; polyethylene glycol, 1 capful/8 oz water PO BID; lactulose, 30 mL PO BID-QID OR 60 mL daily; sorbitol, 30 mL PO q2h x 3, then PRN; magnesium hydroxide, 30–60 mL PO daily-BID; or magnesium citrate, 8 oz PO daily  • **Opioid-induced constipation:** Consider methylnaltrexone, 8 or 12 mg/dose SC, no more than once a day; linaclotide, 72–145 mcg/d PO; naloxegol, 12.5–25 mg/d PO  Not for post-op ileus and mechanical bowel obstruction |
|  | **Diarrhea Life Expectancy: Years; Year to Months; and Months to Weeks** |
|  | **Grade 1**  • Loperamide, 4 mg PO x 1 then 2 mg PO after each loose stool, up to 16 mg/d  • Consider addition of **bulk-forming agents** with caution in patients on opioids  • **If not on opioids:** Diphenoxylate/atropine 1–2 tabs PO q6h PRN, maximum 8 tabs/d Grade 2  • **Initiate/continue loperamide**, 4 mg PO x 1 then 2 mg PO after each loose stool, up to 16 mg/d  • **If not on opioids**: Diphenoxylate/atropine 1–2 tabs PO q6h PRN, maximum 8 tabs/d  • Consider hyoscyamine, 0.125 mg PO/ODT/SL q4h PRN, max: 1.5 mg/d; atropine 0.5–1 mg SC/IM/IV/SL q 4–6h PRN  • **C. diff-induced:** Metronidazole, 500 mg PO/IV QID x 10–14 days; vancomycin, 125–500 mg PO QID x 10–14 days  • **Non-C. diff infection:** Treat appropriately based on culture findings  • **Immunotherapy-related:** Dexamethasone, 4–8 mg/d; infliximab, 5 mg/kg q 2–6 weeks  Grades 3/4 (Inpatient hospitalization w/ICU for Grade 4)  • **Initiate/continue loperamide**, 4 mg PO x 1 then 2 mg PO after each loose stool, up to 16 mg/d  • **If not on opioids:** Diphenoxylate/atropine, 1–2 tabs PO q6h PRN, maximum 8 tabs/d  • Consider hyoscyamine, 0.125 mg PO/ODT/SL q4h PRN, max: 1.5 mg/d; atropine 0.5–1 mg SC/IM/IV/SL q 4–6 h PRN  **• Consider octreotide**, 100–200 mcg/d SC, q8h or by continuous infusion |
|  | **Diarrhea Life Expectancy: Weeks to Days (dying patient)** |
|  | - **Reevaluate** ongoing antidiarrheal, anticholinergic agents   • **Initiate or increase dose** of around-the-clock opioid  • Atropine 0.5–1 mg SC/IV/SL q 4–6h PRN  • Consider octreotide, 100–200 microgram SC q8h   - Consider glycopyrrolate, 0.2–0.4 mg IV q4h PRN |
|  | **Malignant Bowel Obstruction Life Expectancy: Years; Year to Months; Months to Weeks; and Weeks to Days (dying patient)** |
|  | •**Reduce opioid dose or rotate opioid**  •**Metoclopramide**, 5–10 mg PO QID 30 min before meals and at bedtime; avoid in the setting of complete obstruction  •**Dexamethasone**, 4–12 mg IV daily, discontinue if no improvement in 3–5 days  •**Scopolamine** (patch or IV); hyoscyamine, 0.125 mg PO/ODT/SL q4h PRN; glycopyrrolate, 0.2–0.4 mg IV q4h PRN  •**Octreotide**, 100–300 mcg SC BID-TID or 10–40 mcg/h continuous SC/IV infusion; if prognosis >8 weeks, consider long-acting release (LAR) or depot injection |
|  | **Sleep/Wake Disturbance Life Expectancy: Years; Year to Months; and Months to Weeks** |
|  | **Insomnia**  • Trazodone, 25–100 mg PO at bedtime  • Olanzapine, 2.5–5 mg PO at bedtime  • Zolpidem, 5 mg PO at bedtime  • Mirtazapine, 7.5–30 mg PO at bedtime  • Chlorpromazine, 25–50 mg PO at bedtime  • Quetiapine, 12.5–25 mg PO at bedtime  • Lorazepam, 0.5–1 mg PO at bedtime  • For phase shift disorder consider ramelteon (8 mg PO 30 min before bedtime) or melatonin (30 min before bedtime; dosage may vary  by formulation) Daytime Sedation  • Caffeine, 100–200 mg PO q 6 h, last dose 4 PM  • Methylphenidate, start with 2.5–20 mg PO BID, second dose no later than 6 h before bedtime  • Dextroamphetamine, 2.5–10 mg PO BID, second dose no later than 12 h before bedtime  • Modafinil, 100–400 mg PO each morning  **Restless Legs Syndrome (RLS)**  • Ropinirole, 0.25 mg PO 1–3 h before bedtime  • Pramipexole, starting dose 0.125 mg PO at bedtime, may require titration  May also consider pregabalin, carbidopa-levodopa, or low-dose methadone with dopamine agonist; however, all of these medications are off-label for RLS |
|  | **Sleep/Wake Disturbance Life expectancy: Weeks to Days (dying patient)** |
|  | •Titrate dose of existing pharmacotherapy |
|  | **Delirium Life Expectancy: Years; Year to Months; and Months to Weeks** |
|  | **Mild/Moderate Delirium**  • Haloperidol, 0.5–2 mg PO BID/TID  • Alternatives: risperidone, 0.5–2 mg PO BID; olanzapine, 5–20 mg PO daily; or quetiapine fumarate, 25–200 mg PO/SL BID  **Severe Delirium (agitation)**  • Haloperidol, 0.5–2 mg IV q1–4h PRN  • Alternatives: olanzapine, 2.5–7.5 mg PO/SL q2–4h PRN (maximum = 30 mg/d); chlorpromazine, 25–100 mg PO/PR/IV q4h PRN  for bedbound patients  • 4 High-dose neuroleptic-refractory: Consider adding lorazepam, 0.5–2 mg SC/IV q4h |
|  | **Delirium Life Expectancy: Weeks to Days (dying patient)** |
|  | • **Upward titrate** haloperidol, risperidone, olanzapine  • **High-dose neuroleptic-refractory**: Upward titrate lorazepam  • Consider rectal or IV haloperidol  • Consider chlorpromazine, 25–100 mg PO/PR at bedtime with or without lorazepam, 0.5–2 mg SC/IV q6h |
|  | **Palliative Sedation Imminently dying patient** |
|  | • Midazolam or lorazepam, continuous infusion  • Propofol, continuous infusion |
|  | **Recommendations** |
|  | For acute progressive dyspnea or for patients who are not opioid naive, more aggressive titration may be required |
|  | The addition of benzodiazepines to opioids can increase the risk of respiratory depression |
|  | An around-the-clock dosing schedule may provide the most consistent benefit to the patient. |
|  | Continuous intravenous or subcutaneous infusions of different antiemetics may be necessary for the management of intractable NV |
|  | Plain film radiography may be helpful in confirming the clinical diagnosis of bowel obstruction. Consider a CT scan if surgical intervention is contemplated, as it is more sensitive and may help identify the cause of obstruction. |
|  | Most malignant bowel obstructions are partial, allowing time to discuss appropriate intervention with the patient/family/caregiver |
|  | Patient's values and preferences and any decisions should be documented in the medical record, including MOLST/POLST (Medical Orders for Life-Sustaining Treatment or Physician Orders for Life-Sustaining Treatment). |

**ACRONYMS AND ABBREVIATIONS**

| BM | Bowel movement | PRN | As needed |
| --- | --- | --- | --- |
| BID | Twice a day | PR | Prothrombin ratio |
| CNS | Central Nervous System | PO | By mouth |
| CT | Computerized Tomography | PPS | Palliative Performance Scale |
| ESAS | Edmonton Symptom Assessment System | POLST | Physician Orders for Life-Sustaining Treatment |
| GERD | Gastroesophageal reflux disease | QID | Four times a day |
| IM | Intra- Muscular | q2,4,8h | Every 2,4,8 hours |
| IV | Intra-venous | RT | Respiratory therapist |
| MOLST | Medical Orders for Life-Sustaining Treatment | RLS | Restless Legs Syndrome |
| NV | Nausea & Vomiting | SC | Sub-cutaneous |
| NCCN | National comprehensive cancer network | SL | Sub lingual |
| ODT | orally disintegrating tablet | TID | Thrice a day |

**References:**

1. Dans M, Kutner JS, Agarwal R, Baker JN, Bauman JR, Beck AC, et al. NCCN Guidelines® Insights: Palliative Care, Version 2.2021: Featured Updates to the NCCN Guidelines. Journal of the National Comprehensive Cancer Network. 2021;19(7):780-8.
